# m^6^A methyltransferase METTL16 mediates immune evasion of colorectal cancer cells via epigenetically regulating PD-L1 expression

**DOI:** 10.18632/aging.204980

**Published:** 2023-08-29

**Authors:** Ailei Wang, Yingjie Sun, Xince Wang, Zhaofei Yan, Dongsheng Wang, Li Zeng, Qingge Lu

**Affiliations:** 1 Department of Anorectal, Tangshan Traditional Chinese Medicine Hospital, Tangshan, China; 2 Tangshan Traditional Chinese Medicine Hospital, Tangshan, China

**Keywords:** colorectal cancer, epigenetic regulation, N6-methyladenosine, METTL16, PD-L1

## Abstract

Background: The immune checkpoint inhibitors (ICIs) has dramatically changed the therapeutic area of cancers. A great number of patients with CRC exhibit poor response rate to ICI treatment. N6-methyl adenosine (m6A) is closely correlated with the initiation and progression of cancers. To explore the role of methyltransferase-like 16 (METTL16) in CRC treatment.

Methods: Clinical samples and different CRC cell lines were collected. The expression of METTL16 and PD-L1 was determined by qPCR, IHC. Ectopic expression of METTL16 was performed in CRC cells. A co-culture system was established using CRC cells and T cells to measure the immune evasion. Cell viability, apoptosis, migration, and invasion were examined by CCK-8, colony formation, flow cytometry, Transwell, and wound healing assay, respectively. The N6-methyl adenosine (m6A) modification of PD-L1 by METTL16 was investigated by methylated RIP (MeRIP) and RNA stability experiment. *In vivo* xenograft model was established to measure the effects of METTL16 on CRC growth.

Results: METTL16 was decreased and PD-L1 was increased in CRC tissues and cell lines. METTL16 enhanced cell proliferation, migration, and invasion, and promoted CRC tumor growth *in vivo*. METTL16 induced m6A modification and decreased the stability of METTL16 RNA, leading to the suppression of METTL16 level. METTL16 overexpression in CRC cells induced decreased portion of PD-1 positive T cells. Overexpression of METTL16 and inhibition of PD-1 synergistically suppressed *in vivo* growth of CRC cells.

Conclusions: Our work identified the METTL16/PD-L1/PD-1 regulatory axis in CRC development and immune evasion, which represented a promising target for CRC treatment.

## INTRODUCTION

Immune checkpoint inhibitors (ICIs), particularly anti-programmed death receptor 1 (PD-1) therapies, have dramatically changed the face of cancer treatment in recent years [1–4]. Unfortunately, in colorectal cancer (CRC), one of the top three most malignant and prevalent cancers, the anti-tumor effect of immune checkpoint inhibitors (ICIs) is limited to those with high microsatellite instability (MSI-H) [5–8]. As for the microsatellite stable (mSS) CRC patients (approximately 90% of the cases), the response rate is only 5% to 10% [9–11]. A recent clinical report, the REGONIVO trial, indicated that combined treatment with Regorafenib with anti-PD-1 antibody achieved an objective response rate of 33% in CRC patients [12]. However, there is still an urgent need to explore the resistance mechanisms and improve the efficacy of immune checkpoint inhibitors in CRC due to the limited size of current clinical practice and insufficient evidence [13].

Traditional epigenetic regulation refers to the chemical modification of DNA or histones that can regulate gene expression independently of genome sequence changes [14, 15]. Studies have widely exposed the profound effect of dysregulated epigenetic modification enzymes on human diseases including cancers [16–19]. Similar with the epigenetic regulation of DNAs, RNAs also carry hundreds of different sites for different post-transcriptional modifications [17]. N6-methyl adenosine (m6A) is the most important mRNA modification in eukaryotic cells [20]. Mechanistically, m6A modification is a reversible chemical process controlled dynamically by the balanced activity of m6A methyltransferase and demethylase [21]. Since m6A modification has been shown to play a crucial role in RNA translation, stability, and variable splicing, perturbation of the m6A component has been indicated as regulatory factors of human disease, particularly cancer [22]. Dysregulation of the m6A “write” protein, such as the methyltransferase-like 3 (METTL3) and methyltransferase-like 16 (METTL16), has been reported in the disease development in recent years [23, 24]. Nevertheless, the role of METTL16 in immune regulation of CRC has not been clarified yet.

In this work, we explored the correlation between METTL16 with PD-L1 expression and determined the effects of METTL16 on CRC growth and immune evasion. Our findings may provide novel target for the treatment of CRC.

## MATERIALS AND METHODS

### Evaluation of clinical samples

Patients diagnosed with CRC (n=20) and hospitalized in Tangshan Traditional Chinese Medicine Hospital were recruited in this study. Tumor and adjacent non-tumor tissues were collected during surgical operation and made into paraffin-embedded samples or frozen into liquid nitrogen for further experiments. All patients have signed the informed consents.

### Xenograft model

Male BALB/c nude mice that aged 4 to 6-week-old were bought from Vital River Laboratory (China). SW480 cells transfected with control or METTL16 overexpression vectors (5 x 10^6^ cells/site) were suspended in 50 μl saline and inoculated into the fact pat on back. Tumor size was measured and calculated every three days. After feeding for 30 days, the tumors were collected and made into paraffine-embedded samples.

### IHC staining

Paraffine-embedded tissues were dewaxed and blocked with goat serum. After incubation with primary antibody against PD-L1 (1:200, Abcam, USA), METTL16 (1:200, Abcam, USA), and Ki-67 (1:200, Abcam, USA), the samples were probed with HRP-conjugated secondary antibodies and reacted with DAB for visualization. Images were taken using a light microscope (Leica, Germany).

### Cell lines and transfection

CRC cell lines NCM460, RKO, HCT116, SW480, Colo320, DLD-1, HCE8693, SW620, and HT29, and the normal human colon epithelial cell line FHC were purchased from Wuhan Procell and ATCC. NCM460, DLD-1, HCE8693, and Colo320 were maintained in RPMI-1640, RKO in MEM, HCT116 and HT29 in McCoy’s 5A, SW620, SW480 in Leibovitz’s L-15 medium that supplied with 10% FBS (BI, Israel) and 1% penicillin and streptomycin (Sigma, USA) and maintained in 37° C incubator with 5% CO_2_.

METTL16 proteins siRNAs and overexpression vectors were obtained from GenePharma (Shanghai, China). Cells were transfected with indicated oligonucleotides using the Lipofectamine 2000 reagent (Thermo, USA) as per manufacturer’s protocol. Transfection efficacy was examined 2 days after transfection.

### Cell viability and proliferation detection

Cell viability was measured by cell counting kit-8 (CCK-8, SolarBio, China) following the manufacturer’s protocol. CRC cells were seeded into 96-well plate with 5,000 cells per well and incubated at 37° C incubator for indicated time points. Then CCK-8 reagent was added and incubated for 2 hours. The absorbance values at 450 nm were measured by a microplate detector. For colony formation assay, cells were seeded into 6-well plate and incubated for 10 days. The visible colonies were then stained with crystal violet for 20 min. Images were taken by a digital camera.

### Cell apoptosis

Cell apoptosis was measured using Annexin V/PI apoptosis detection kit (Beyotime, China) in line with manufacturer’s introduction. In brief, cells were collected and suspended in binding buffer that contains Annexin V and PI reagent. After incubation for 30 minutes in dark, the samples were loaded in a flow cytometer (BD Bioscience, USA) and the portion of apoptotic cells were measured.

### Transwell assay

CRC cells were suspended in serum-free medium and seeded into the upper chambers of Transwell (Costar, USA). The lower chambers were filled with complete culture medium. After incubation for 24 hours, the upper chambers were collected, and the inner side of chamber was wiped with cotton sticks. The migrated cells were then stained with crystal violet for 20 min. To measure cell invasion, the Transwell chambers were pre-coated with Matrigel that mixed with Leibovitz’s L-15 at 1:1. Images of cells were taken under a microscope (Leica, Germany) [25].

### Wound healing experiment

CRC cells were seeded into 6-well plate and cultured overnight to confluence. The monolayer of cells was then scratched with a sterile 200 μl pipette and washed with PBS to remove debris. Cells were then incubated for 24 hours, and images of the wounds were taken at 0. 24, and 48 h after scratching.

### Western blotting assay

Cell pellets were homogenized with RIPA lysis buffer and quantified with BCA kit. Protein samples containing 40 mg of protein were separated by SDS-PAGE gel and transferred onto PVDF membranes, followed by blocking in 5% skim milk for 2 hours. The blots were then probed with primary antibodies that target E-cadherin (1:2000, ab231303, Abcam, USA), Vimentin (1:2000, ab92547, Abcam, USA), METTL16 (1:2000, ab252420, Abcam, USA), and β-actin (1:2000, ab8226, Abcam, USA) overnight at 4° C. The membranes were then hatched with HRP-conjugated secondary antibodies and ECL reagent for visualization.

### Quantitative real time PCR (qPCR)

Total RNAs were extracted using the Trizol solution (Qiagen, USA). Then a total of 1 μg RNA was subjected to reverse transcription reactions using a Reverse Transcription kit (Qiagen, USA). PCR amplification was conducted using the SYBR GREEN qPCR mix (Thermo, USA).

PD-L1 forward primer: 5’- TGGCATTTGCTGAACGCATTT-3’,

PD-L1 reverse primer: 5’-TGCAGCCAGGTCTAATTGTTTT-3’;

METTL16 forward primer: 5’-CTCTGACGTGTACTCTCCTAAGG -3’,

METTL16 reverse primer: 5’- TACCAGCCATTCAAGGTTGCT-3’.

### RNA stability detection

To detect the stability of METTL16 mRNA, CRC cells were treated with α-amanitin (50 μM; Sigma, USA) for indicated hours. Then total RNA was extracted and reverse transcribed. The relative level of METTL16 RNA to 0 hour was measured by qPCR assay.

### Protein degradation

CRC cells were treated with the protein synthesis inhibitor cycloheximide (CHX) for 0, 3, 6, 12 hours, then homogenized to collect total protein. The expression of PD-L1 was detected by western blot assay.

### RNA immunoprecipitation (RIP)

The enrichment of METTL16 on PD-L1 RNA was measured by using EZ-Magna RIP Kit (Millipore, USA) as per manufacturer’s instruction. In brief, magnetic beads were pre-coated with anti-METTL16 antibody overnight at 4° C in rotation. Next day, the antibody-beads conjugates were hatched with the isolated total RNAs for 6 hours at 4° C. The precipitated RNAs were then eluted and purified. The level of PD-L1 was measured by qPCR.

### MeRIP-qPCR assay

The RNA was collected from cells and were hatched with anti-m6A antibody (Thermo, USA) for 6 hours at 4° C. The Dynabeads (Invitrogen, USA) were blocked by 2% BSA (Thermo, USA) for 3 hours at 4° C, followed by incubation with aforementioned RNA-antibody complex at 4° C overnight. After elution with SDS buffer, the RNAs were collected and level of PD-L1 was quantified by with qPCR assay [26].

### T cell activation and co-culture system

Human peripheral blood samples were obtained from donors and the peripheral blood mononuclear cells (PBMCs) were purified by Ficoll methods. For T cell activation, PBMCs were incubated with Dynabeads Human T-Activator CD3 and CD28 (Gibco, USA) in line with the manufacturer’s protocol for 3 days. For co-culture, T cells and CRC cells were placed in the upper and lower chambers of Transwell, respectively. The ratio of T cells and cancer cells was 10:1 and cultured for 3 days. To determine the activation of T cells, PMSCs were incubated with anti-CD4, anti-CD-8, and anti-PD-1 antibody and analyzed by a flow cytometer.

### Statistics

All statistical analyses were performed using the SPSS and GraphPad Prism 7.0 Software. The comparison between experimental groups was conducted using two-sided Student’s t-test and one-way ANOVA followed by Dunnett’s multiple comparison. P < 0.05 was set as significant.

## RESULTS

### Expression of METTL16 and PD-L1 in clinical samples and cell lines of CRC

**Figure 1:**
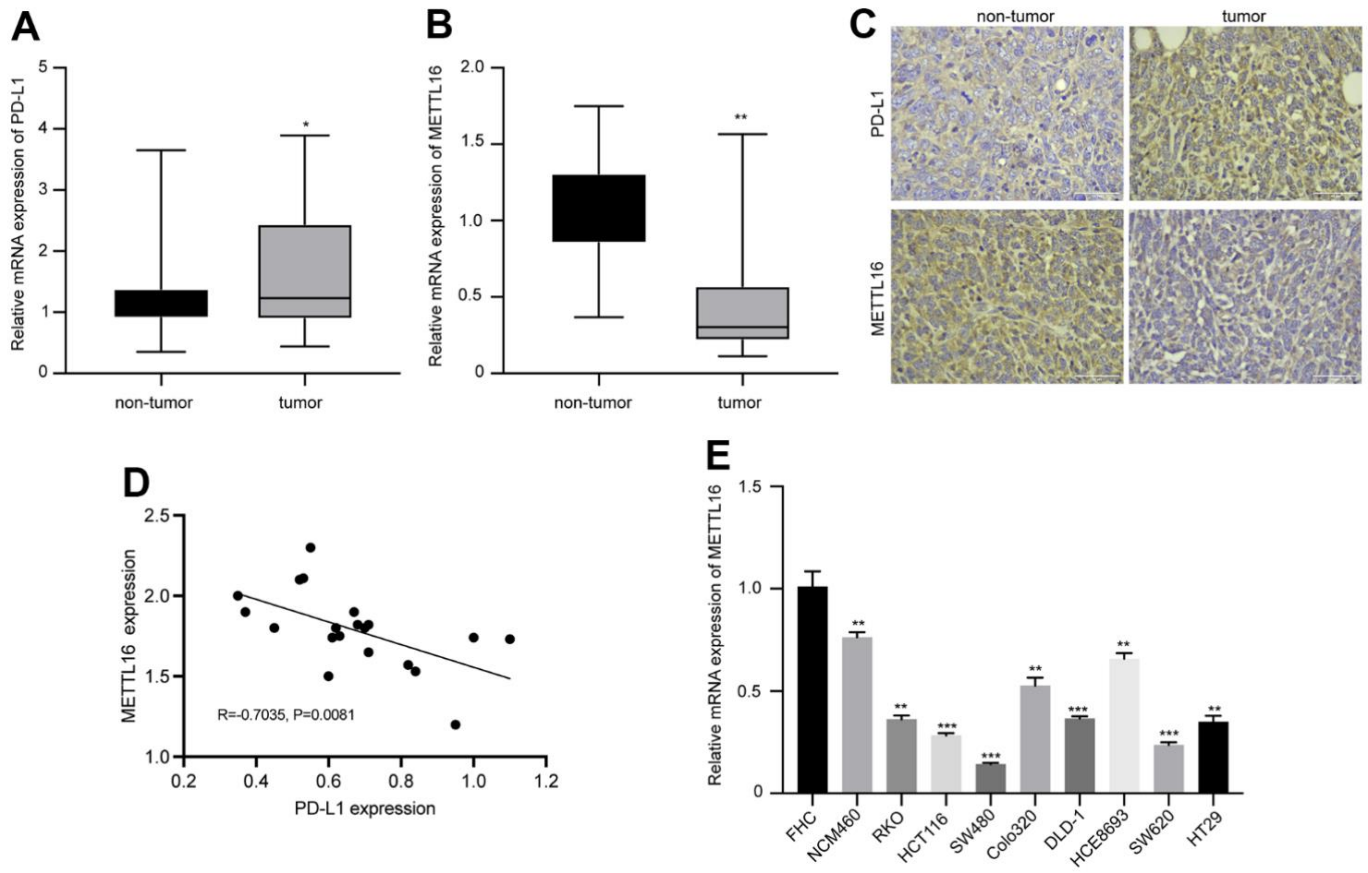
**Expression of METTL16 and PD-L1 in clinical samples and cell lines of CRC.** CRC tumor and non-tumor tissues were collected from patient with CRC. The RNA levels of (**A**) PD-L1 and (**B**) METTL16 were measured by qPCR assay. (**C**) The protein expression of PD-L1 and METTL16 was measured by IHC experiment. (**D**) The Pearson correlation analysis of PD-L1 and METTL16 in tumor tissues (n=20). (**E**) The RNA level of METTl16 in CRC cell lines (NCM460, RKO, HCT116, SW480, Colo320, DLD-1, HCE8693, SW620, and HT29) and normal human colon epithelial cell line FHC was measured by qPCR. *p<0.05, **p<0.01, ***p<0.001.

We first analyzed the expression and correlation of METTL16 and PD-L1 in CRC. As shown in Figure 1A, 1B, the RNA level of PDL-1 in collected CRC tumor tissues is notably elevated, whereas the level of METTL16 is significantly decreased, compared with the non-tumor tissues. The IHC analysis of protein expression also indicated elevated PD-L1 and suppressed METTL16 expression in CRC tumor tissues compared with the paired non-tumor tissues (Figure 1C). Results from qPCR indicated a negative correlation between expression of METTL16 and PD-L1 (R=0.7035, p=0.0081). Moreover, we observed significantly decreased level of METTL16 in CRC cell lines, including NCM460, RKO, HCT116, SW480, Colo320, DLD-1, HCE8693, SW620, and HT29, compared with the normal human colon epithelial cell line FHC (Figure 1E). These data indicated that METTL16 may be negatively correlated with PD-L1 expression in CRC.

### METTL16 regulates the proliferation, migration and invasion of CRC cells

**Figure 2:**
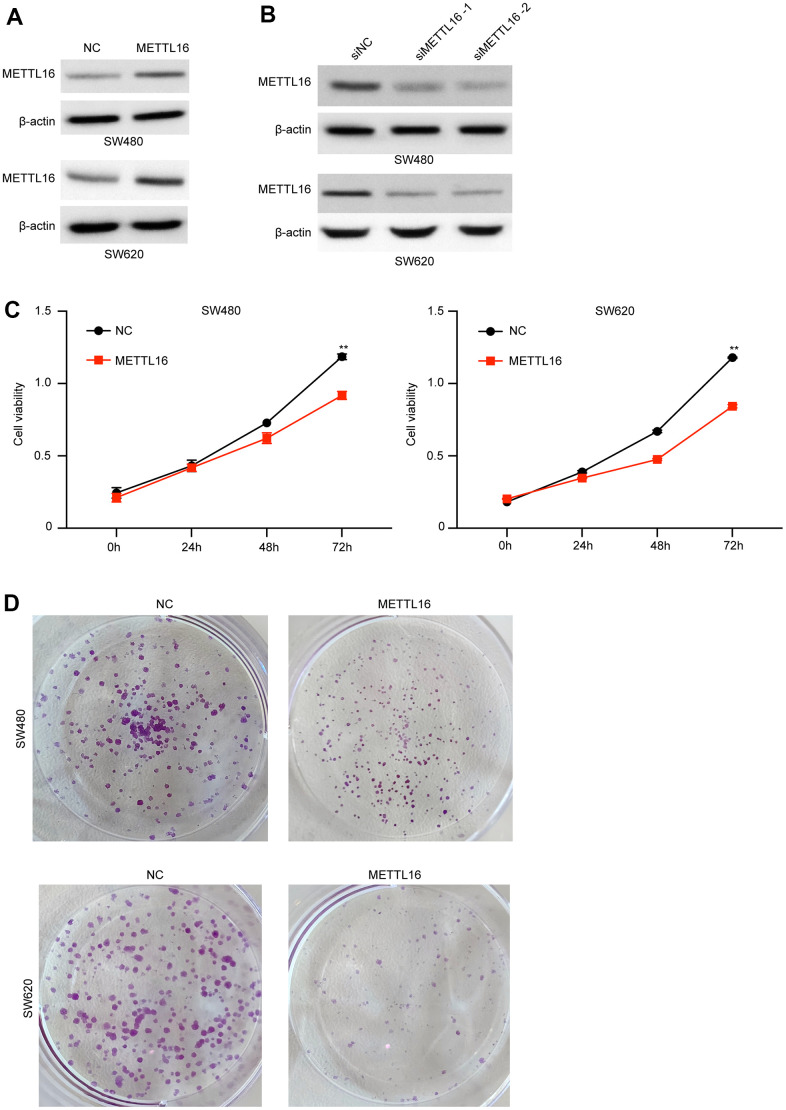
**METTL16 regulates the proliferation of CRC cells.** (**A**) SW480 and SW620 cells were transfected with METTL16 overexpression vectors, and protein level of METTL16 was measured by western blotting assay. (**B**) SW480 and SW620 cells were transfected with METTL16 siRNAs, then protein level of METTL16 was measured by western blotting assay. (**C**) Cell viability of SW480 and SW620 cells under overexpression of METTL16 was measured by CCK-8 assay. (**D**) Proliferation of SW480 and SW620 cells under overexpression of METTL16 was detected by using colony formation assay. **p<0.01.

**Figure 3:**
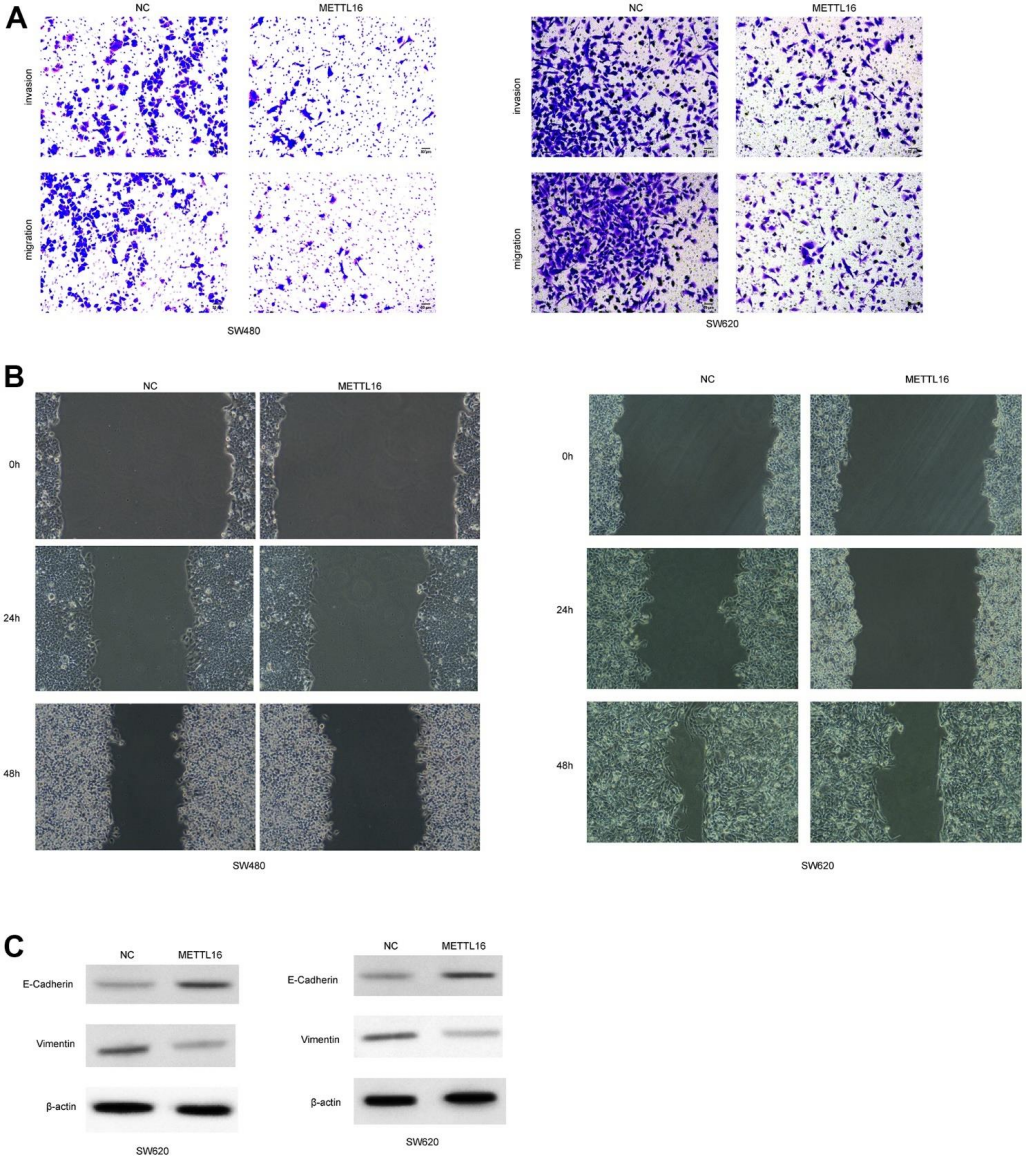
**METTL16 regulates the migration and invasion of CRC cells.** SW480 and SW620 cells were transfected with METTL16 overexpression vectors. (**A**) Cell migration and invasion were detected by using Transwell experiment. (**B**) Cell migration was measured by wound healing assay. (**C**) Protein levels of E-cadherin and Vimentin were detected by western blotting assay.

Moreover, among the included CRC cell lines, the SW480 and SW620 exhibited the lowest level of METTL16 and hence were selected for further experiments. We subjected transfection of ectopic expression vectors and siRNAs of METTL16 in these two cell lines and assess cell proliferation, migration, and invasion. The results from western blotting assay demonstrated the successful overexpression and depletion of METTL16 in SW480 and SW620 cells (Figure 2A, 2B). As shown in Figure 2C, 2D, overexpression of METTL16 led to significantly suppressed growth (Figure 2C) and colonies (Figure 2D) of SW480 and SW620 cells. Besides, results from Transwell assay indicated that overexpression of METTL16 notably suppressed the number of migrated and invaded cells (Figure 3A). The suppressed healing of wounds in Figure 3B also suggested the impeded cell migration. Results from western blotting showed that overexpression of METTL16 caused increased level of E-cadherin and decreased level of Vimentin in SW480 and SW620 cells (Figure 3C), indicating the enhanced EMT. These data indicated that METTL16 suppressed CRC cell proliferation, migration and invasion.

### METTL16 modulates immune invasion in a CRC cell/T cell co-culture system

**Figure 4:**
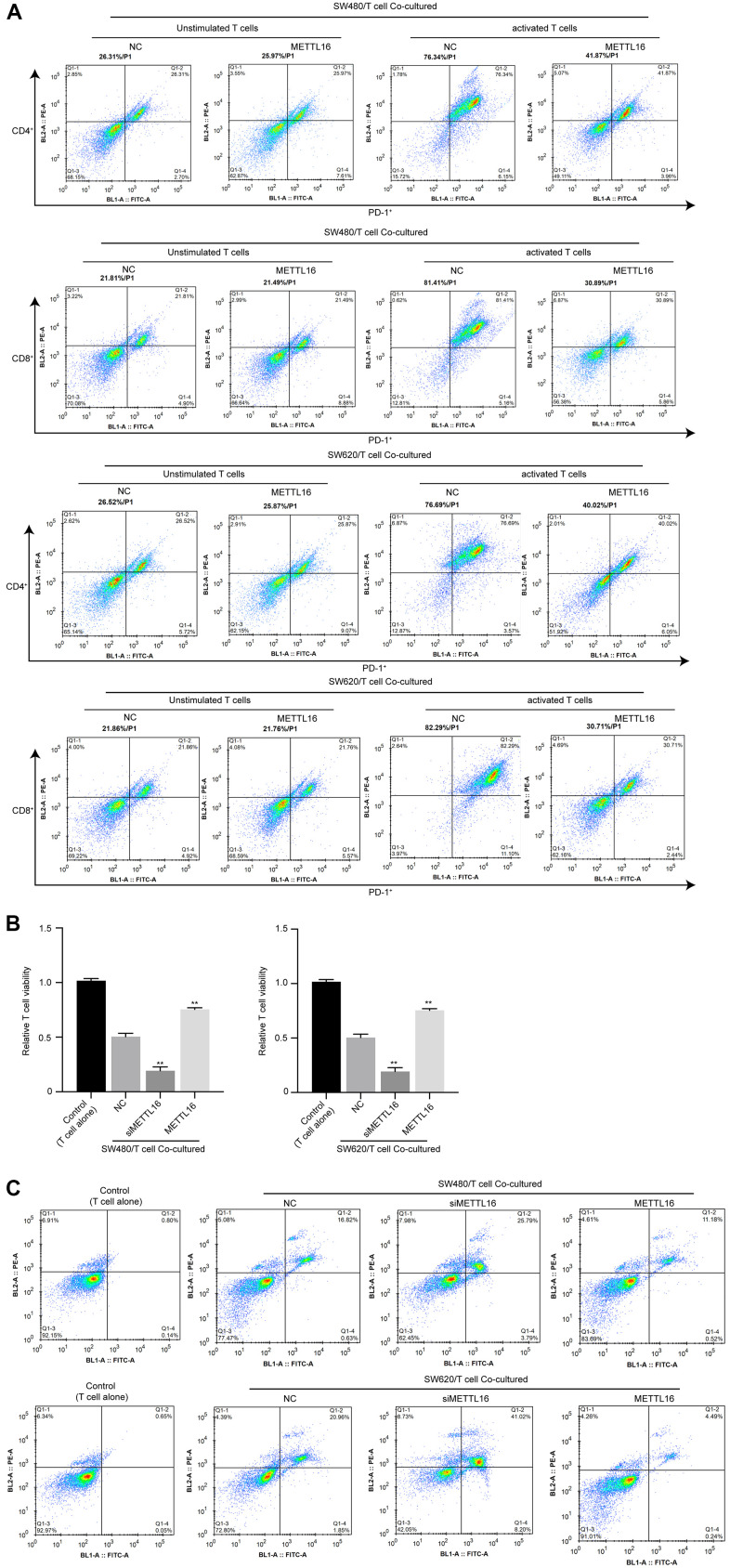
**METTL16 modulates immune invasion in a CRC cell/T cell co-culture system.** CRC cells were co-cultured with unstimulated or activated T cells. (**A**) The portion of PD-1+/CD4+ T cells and PD-1+/CD8+ T cells was measured by flow cytometry. (**B**) Cell viability was assessed by CCK-8 assay. (**C**) Cell apoptosis was measured by flow cytometry. **p<0.01.

To explore the effects of METTL16 on immune invasion of CRC cells, we adopted a co-culture system of CRC cells with unstimulated or activated T cells and evaluated the activation, viability and apoptosis of T cells. As shown in Figure 1A, the portions of PD-1+ cell in activated T cells were notably decreased under co-culture with METTL16-overexpressed CRC cells, compared with those co-cultured with control CRC cells. Moreover, co-culture with CRC cells notably suppressed the viability and induced apoptosis of T cells (Figure 4B, 4C), which were significantly enhanced by depletion of METTL16 in CRC cells. Besides, overexpression of METTL16 reversed the suppressive effects of CRC cells on T cell viability (Figure 4B, 4C). These data suggested that METTL16 overexpression in CRC cells could promote immune response of T cells.

### METTL16-mediated m^6^A modification regulates PD-L1 expression in CRC cells

**Figure 5:**
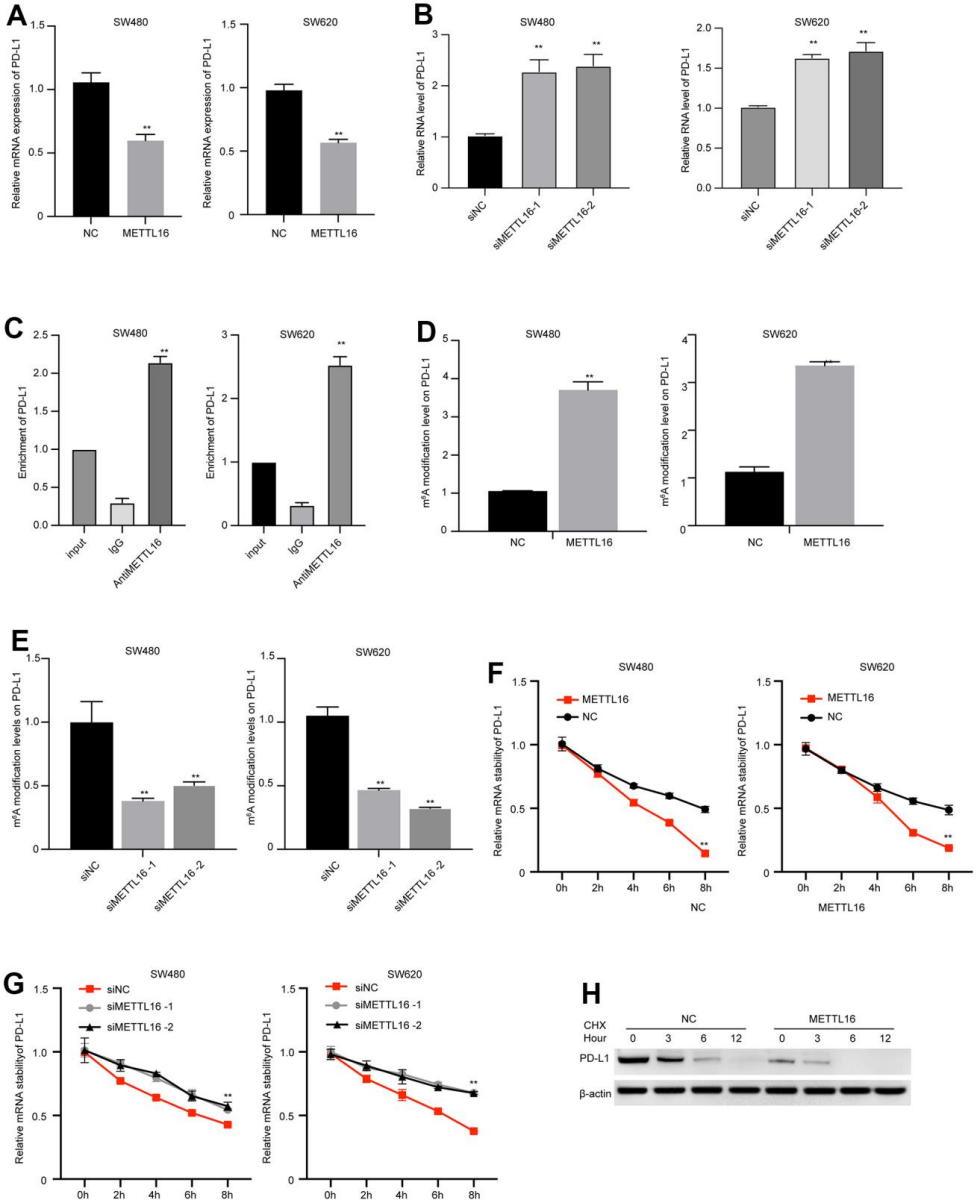
**METTL16-mediated m^6^A modification regulates PD-L1 expression in CRC cells.** (**A**, **B**) SW480 and SW620 cells were transfected with METTL16 overexpression vectors or siRNAs, then RNA level of PD-L1 was measured by qPCR assay. (**C**) The interaction between METTL16 with PD-L1 mRNA was measured by RIP assay. (**D**, **E**) SW480 and SW620 cells were transfected with METTL16 overexpression vectors or siRNAs, and MeRIP assay was conducted to assess m^6^A enrichment. (**F**, **G**) SW480 and SW620 cells were transfected with METTL16 overexpression vectors or siRNAs and treated with RNA synthesis inhibitor α-amanitin (50 μM). The stability of PD-L1 mRNA was checked by qPCR. (**H**) SW480 cells were treated with CHX for indicated time points and the expression of PD-L1 was measured by western blot. **p<0.01.

METTL16 is a well-recognized regulator of RNA methylation. Hence, we wondered if METTL16 directly modulates the expression of PD-L1. We observed that overexpression of METTL16 caused significant decrease in PD-L1 mRNA level (Figure 5A), and depletion of METTL16 markedly increased PD-L1 mRNA level (Figure 5B). We validated the elevated m6A modification level in several reported target of METTL16, including the RAB11B-AS1, MAT2A, and EEF1A1 (Supplementary Figure 1). Consistent with these findings, the results from RIP experiment indicated that the PD-L1 mRNA was modified by m6A (Figure 5C). Simultaneously, the m^6^A level of PD-L1 was upregulated by METTL16 overexpression and downregulated by METTL16 knockdown (Figure 5D, 5E), suggesting that METTL16 regulated the methylation of PD-L1 in CRC cells. Moreover, m6A modification has been reported to modulate RNA stability. Here, we examined the PD-L1 RNA stability after using α-amanitin to block new RNA synthesis. The degradation of PD-L1 mRNA was notably faster in METTL16-overexpressed cells, compared with the control cells (Figure 5F), and depletion of METTL16 maintained the stability of PD-L1 mRNA (Figure 5G). Moreover, treatment with Cycloheximide (CHX), the protein synthesis inhibitor, led to degradation of PD-L1 expression, and overexpression of METTL16 enhanced the PD-L1 degradation (Figure 5H). These data indicated that METTL16 could induce the m^6^A modification of PD-L1 mRNA and decreased its stability.

### METTL16 modulates CRC cell growth *in vivo*

**Figure 6:**
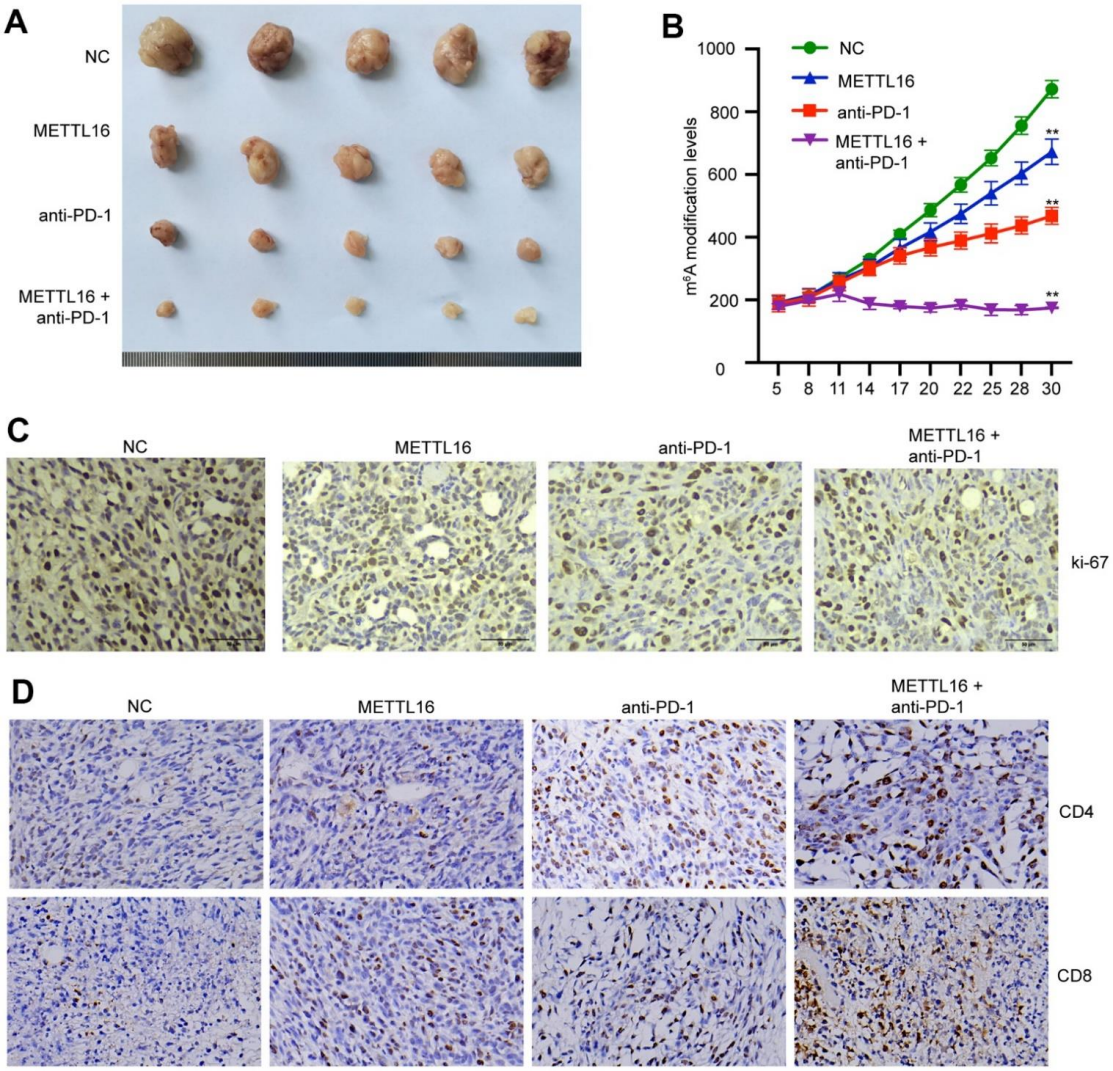
**METTL16 modulates CRC cell growth *in vivo*.** The xenograft mouse model was established using control and METTL16 overexpressed SW480 cells and treated with anti-PD-1 antibody. (**A**) Images of tumors were taken. (**B**) Tumor growth curve was recorded. (**C**) The expression of Ki-67 was measured by IHC assay. (**D**) The expression of CD8 and CD4 in tumor tissues was measured by IHC assay. **p<0.01.

We next investigated the *in vivo* effects of METTL16 on CRC development. A xenograft tumor model was established using SW480 cells and treated with anti-PD-1 antibody. We observed that overexpression of METTL16 suppressed the tumor size and tumor growth curve and enhanced the tumor suppressive effect of anti-PD-1 antibody (Figure 6A, 6B). Analysis on tumor tissues demonstrated that overexpression of METTL16 and blocking of PD-1 could notably suppress the expression of proliferative biomarker Ki-67 (Figure 6C). Moreover, the levels of CD4 and CD8 positive cells in tumor tissues were notably elevated by overexpression of METTL16 and anti-PD-1 treatment compared with the control group and were further increased by their combination (Figure 6D).

## DISCUSSION

The involvement of m^6^A modification has been reported in various pathophysiological processes, and its aberrant regulation is correlated with the initiation and development of a variety of diseases [27]. The representative m6A methyltransferases and demethylases, such as ALKBH5, METTL3, and METTL14, have been widely studied in cancer development [27]. The correlation between METTL16 with cancers also draws great attention in recent years [28, 29]. The level of METTL16 is upregulated in glioma [30] and is correlated with poor prognosis of melanoma [31]. Wang et al. reported that METTL16 promotes the proliferation of gastric cancer cells via upregulating the expression of cyclin D1 [27]. In this study, we observed decreased level of METTL16 in clinical CRC tumor samples and CRC cell lines, compared with the non-tumor tissues and normal cell line. Further *in vitro* experiments revealed that overexpression of METTL16 led to suppressed CRC cell growth, migration, and invasion.

It has been demonstrated that METTL16 could function in m6A-dependent or -independent manner [32]. For example, METTL16 mediated the translation of CIDEA in a m^6^A-dependent manner and promoted non-alcoholic fatty liver disease [33]. Degradation of MAT2A pre-mRNA mediated by METTL16 was disrupted by oxidative stress, which consequently aggravated the apoptosis of nucleus pulposus cells and exacerbated the process of intervertebral disc degeneration [34]. Here, we identified that overexpression of METTL16 decreased the expression of PD-L1 in CRC cells through inducing the m^6^A modification and degradation of PD-L1 RNA. PD-L1 is the ligand protein produced by tumor cells, which can bind to PD-1 of T lymphocytes [35]. The interaction of PD-L1 with PD-1 consequently inhibits the immune recognition function and the cytotoxicity of T lymphocytes against tumor cells, and therefore facilitates the immune evasion and tumor growth [36, 37]. In this work, we established a CRC cell/T cell co-culture system and revealed that overexpression of METTL16 in CRC cells suppressed the portion of PD-1 positive cells in the activated T cells, implying the decreased immune evasion. The results from *in vivo* experiments presented synergetic tumor suppressive effects of METTL16 overexpression and PD-1 inhibition. Our findings provided novel regulatory mechanism underlying CRC progression and a potential target for its clinical therapy. Nevertheless, certain limitation exists in current study. For example, further investigation of the response of immune system upon METTL16 alteration is needed.

## CONCLUSIONS

To conclude, we identified the suppressed METTL16 expression in CRC tumors and demonstrated that METTL16 overexpression suppressed CRC cell proliferation, migration and invasion. Mechanistically, METTL16 induced the m^6^A modification of PD-L1 to inhibit immune evasion and enhance the therapeutic effects of anti-PD-1 treatment on CRC.

## Supplementary Material

Supplementary Figure 1
