# Supplementary material for: m6A methyltransferase METTL16 mediates immune evasion of colorectal cancer cells via epigenetically regulating PD-L1 expression
Source: Aging (Albany NY). 2023 Aug 29;15(16):8444–57. doi: 10.18632/aging.204980 (PMC10496997; doi:10.18632/aging.204980)
Supplement: Supplementary Figure 1 [file aging-15-204980-s001.pdf]

SUPPLEMENTARY FIGURE

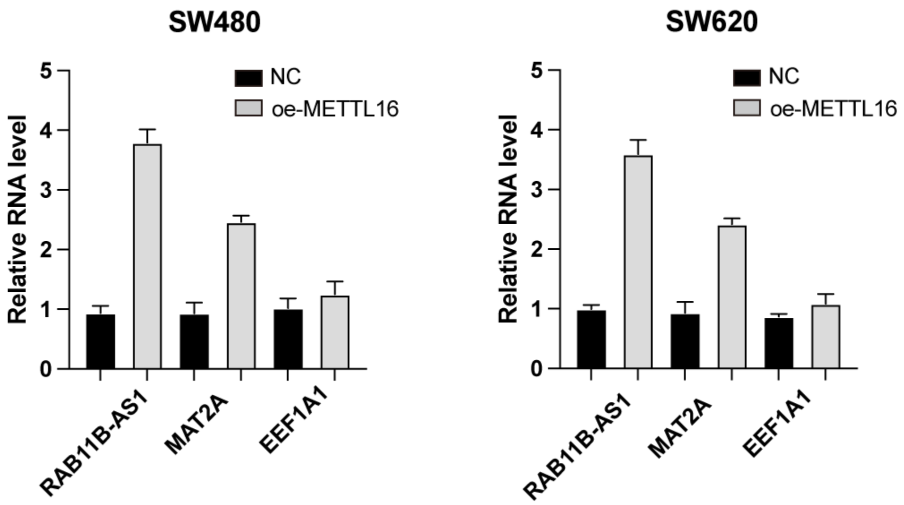

Supplementary Figure 1. The RNA level of RAB11B-AS1, MAT2A, and EEF1A1 in CRC cells under alteration of METTL16.
